# The role of attachment and dissociation in the relationship between childhood interpersonal trauma and negative symptoms in psychosis

**DOI:** 10.1002/cpp.2731

**Published:** 2022-04-06

**Authors:** Amy Degnan, Katherine Berry, Charlotte Humphrey, Sandra Bucci

**Affiliations:** ^1^ Division of Psychology and Mental Health, School of Health Sciences, Faculty of Biology, Medicine and Health Manchester Academic Health Sciences Centre, The University of Manchester Manchester UK

**Keywords:** attachment, childhood trauma, dissociation, negative symptoms, psychosis

## Abstract

Negative symptoms have an adverse impact on quality of life and functioning in psychosis. Service users with psychosis have identified negative symptoms as a priority for their recovery. Despite this, there is a lack of effective and targeted psychological interventions for negative symptoms and their underlying mechanisms remain poorly understood. Childhood trauma is a robust risk factor for positive symptoms in psychosis, but the association with negative symptoms is less well established. Our aim was to examine the association between childhood interpersonal trauma and negative symptoms and the psychological mediators of this relationship. Two hundred and forty participants experiencing psychosis completed validated self‐report measures of childhood trauma, attachment, dissociation, compartmentalization, and symptoms. Mediation analyses showed that disorganized attachment and dissociative experiences mediated the association between childhood trauma and negative symptoms, when analysed individually and in a combined model. Models adjusted for age and positive and depressive symptoms. Avoidant attachment and compartmentalization were independently associated with negative symptoms but not childhood trauma and thus were not significant mediators. Childhood trauma was not independently associated with negative symptoms. This paper is the first to present empirical data to support a model implicating attachment and dissociation as important psychological processes in the link between childhood trauma and negative symptoms. These exploratory findings suggest that it may be beneficial to consider these relationships in trauma‐informed formulations and interventions. Further longitudinal research is required to establish causality and test theoretical models of mechanisms in the pathway to negative symptoms.

**Key practitioner points**

- Disorganized attachment and dissociation may be important psychological mediators in the relationship between childhood interpersonal trauma and negative symptoms in psychosis.
- Attachment patterns and dissociative experiences should be explored in trauma‐informed psychological assessments and formulations and targeted in therapeutic interventions for negative symptoms.
- Dissociative mechanisms may be more important for experiential negative symptoms, involving social withdrawal, avolition, and anhedonia, rather than expressive symptoms, involving diminished emotional expression and alogia.
- A dimensional symptom‐specific approach to assessment and formulation may improve understanding of and inform specialized interventions for negative symptoms in psychosis.

## 1 INTRODUCTION

Negative symptoms are common in psychosis; up to 60% of individuals diagnosed with schizophrenia may have prominent clinically relevant negative symptoms that require treatment (Bobes et al., 2010; Rabinowitz, Berardo, et al., 2013). Negative symptoms are characterized by restricted emotional expression (blunted affect), poverty of speech (alogia), decreased motivation to initiate goal‐directed behaviour (avolition) or social interaction (asociality), and reduced interest and ability to experience pleasure (anhedonia) (Kirkpatrick et al., 2006). Negative symptoms have been overlooked compared with positive symptoms in psychosis, despite being strongly associated with poorer outcomes including wellbeing (Strauss et al., 2012) and functioning (Rabinowitz et al., 2012). Service users have identified negative symptoms as a priority for recovery from psychosis (Sterk et al., 2013). Meta‐analyses of psychosocial interventions for negative symptoms have found that effect sizes are moderate at best, with high heterogeneity across trials (Fusar‐Poli et al., 2014; Lutgens et al., 2017).

There is an ongoing debate about negative symptoms, their origins, and underlying mechanisms (Strauss et al., 2018). Results from factor analyses show that negative symptoms are characterized by two domains: expressive symptoms, including blunted affect and alogia, and experiential symptoms, including avolition, asociality, and anhedonia (Kirkpatrick et al., 2010; Messinger et al., 2011). Certain symptoms within the experiential domain are associated with other mental health problems, particularly depression, whereas those in the expressive domain are specific to psychosis (Krynicki et al., 2018). Depression can be an outcome of negative symptoms or a mechanism by which negative symptoms arise (Hardy, 2017). Differentiating causal pathways underpinning experiential and expressive symptoms in psychosis (Hardy, 2017) is important in developing more individualized therapeutic approaches (Thomas, 2015).

It is now widely accepted that exposure to trauma in childhood, including abuse (physical, sexual, and emotional), neglect, and bullying, increases the risk of developing psychosis (Matheson et al., 2013; van Dam et al., 2012; Varese et al., 2012). Negative symptoms have been conceptualized as an adaptive response to adverse childhood experiences (Griffiths & McLeod, 2020). However, the relationship between childhood trauma and negative symptoms is less well established than positive symptoms (Bailey et al., 2018).

Insecure attachment is one psychological mechanism that has been implicated in the relationship between trauma and negative symptoms in psychosis (Griffiths & McLeod, 2020). Bowlby (1973) proposed that interactions with attachment figures during infancy lead to the development of mental representations of the self in relation to others. These *internal working models* (IWMs) are suggested to manifest as attachment patterns that regulate cognitive, behavioural, and affective responses in future interpersonal relationships. Individuals will develop a secure attachment pattern when they have received warm, sensitive, consistent, and responsive caregiving in childhood (Ainsworth, Blehar, Water, & Wall, 1978). Insecure attachment patterns develop when early caregiving experiences are insensitive, rejecting, neglecting, or unreliable (Ainsworth et al., 1978).

An insecure avoidant attachment pattern is suggested to involve negative IWMs of others as uncaring and unavailable, due to consistent neglect or rejection from caregivers (Bartholomew & Horowitz, 1991). In adulthood, it has been linked to the overregulation and deactivation of affect, interpersonal distancing, and a lack of trusting and confiding relationships (Fraley, 2002). Avoidant attachment is overrepresented in people with psychosis (Gumley, Taylor, et al., 2014) and has been associated with specific negative symptoms, including loss of the ability to experience pleasure (anhedonia; Berry et al., 2007; Berry, Wearden, et al., 2006) and social and emotional withdrawal (Korver‐Nieberg et al., 2015). Attachment insecurity predicts poor recovery from negative symptoms (Gumley, Schwannauer, et al., 2014). Negative symptoms have been suggested to involve deactivation strategies related to attachment avoidance, whereby an individual controls their interpersonal behaviour, for example, through social withdrawal, and inhibits the attachment system in response to threats to self‐security (Griffiths & McLeod, 2020; Liotti & Gumley, 2008).

There has been increasing interest in the role of disorganized attachment in explaining the relationship between trauma and psychosis (Berry et al., 2017). Disorganized attachment is hypothesized to develop because of conflict between the child's attachment and defence systems, whereby the caregiver is perceived to be both the source of and solution to threat (Liotti, 2004). Evidence suggests that childhood trauma, particularly sexual and physical abuse, is strongly related to disorganized attachment (Bucci et al., 2017). Fearful attachment is suggested to be the adult equivalent of disorganized attachment in childhood (Simpson & Rholes, 2002). Empirical research shows that childhood interpersonal trauma is more strongly associated with fearful attachment compared with other attachment patterns in psychosis (Pearce et al., 2017). Fearful attachment is associated with IWMs of the self as unworthy and others as untrustworthy and rejecting (Bartholomew, 1994). This may result in disorganized attachment strategies involving oscillations between approach and avoidance behaviours in relationships, desiring social contact but fearing closeness and rejection (Liotti & Gumley, 2008). Attachment theorists have suggested that, when faced with a stressor in adulthood, disorganized coping responses are activated causing reactions that mirror dissociation, in which an individual is unable to coherently integrate thoughts, feelings, and experiences into consciousness, memory and self‐identity (Liotti, 2004).

Dissociation is recognized as an adaptive response to traumatic experiences (Liotti & Gumley, 2008). Dissociative symptoms are elevated in people with psychosis and trauma‐related diagnoses (Lyssenko et al., 2018), with a particularly robust effect observed for emotional abuse (Rafiq et al., 2018). Researchers have proposed two distinct forms of dissociation thought to have different underlying psychological mechanisms: detachment, characterized by depersonalization and derealization or disconnect from one's body and surroundings, and compartmentalization, including disruptions in normal processes for the monitoring and control of mental experiences, dissociative amnesia, and identity confusion (Brown, 2006; Holmes et al., 2005). These dissociative subtypes may map on to different symptoms in psychosis (Vogel et al., 2013). For example, compartmentalization, but not detachment, was associated with negative symptoms in psychosis, whereas both types of dissociation were related to positive symptoms (Vogel et al., 2013). Meta‐analyses have demonstrated robust positive associations between dissociative experiences and positive symptoms in psychosis (Longden et al., 2020; Pilton et al., 2015). The association between dissociative symptoms and negative symptoms has not been sufficiently explored, though meta‐analytic studies point to a potential relationship: Longden et al. (2020) found small and heterogenous positive associations between dissociation and negative symptoms.

Although theoretical perspectives have highlighted the importance of attachment as a mediator between trauma and negative symptoms (Harder, 2014), only three empirical studies have tested this, with mixed results (Williams et al., 2018). Research into disorganized attachment and psychosis is sparse, and most studies have instead focused on fearful attachment as a proxy measure; a validated self‐report measure of disorganized attachment was only recently developed for people with psychosis (Pollard et al., 2020). Dissociation has been found to mediate the relationship between childhood trauma and voices and paranoia (Pearce et al., 2017). Despite the wealth of research showing that dissociation may be an adaptive response to trauma in psychosis (Rafiq et al., 2018), the mediating role of dissociation in the relationship between trauma and negative symptoms has not been tested and remains poorly understood.

The purpose of this study was to examine the relationship between childhood interpersonal trauma and negative symptoms in psychosis and determine if this relationship was mediated by attachment and dissociation. An exploratory aim was to delineate the specific psychological pathways by which childhood trauma may lead to the abovementioned subdomains of negative symptoms: the expressive subdomain (i.e., reduced emotional range and alogia) and the experiential subdomain (i.e., social withdrawal, avolition, and anhedonia). The following hypotheses were tested: (i) childhood interpersonal trauma would have a direct/total effect on negative symptoms; (ii) disorganized attachment, avoidant attachment, dissociative experiences (combined assessment of detachment, amnesia, and absorption), and compartmentalization would mediate this relationship. Hypotheses were tested in a series of models controlling for the effects of depressive and positive symptoms, given their conflation with negative symptoms (Krynicki et al., 2018).

## 2 METHOD

### 2.1 Design and sample

This was a cross‐sectional correlational design using an online survey. Participants were eligible if they: (i) self‐reported a diagnosis of psychosis, (ii) reported receiving antipsychotic medication or mental health support or treatment for experiences related to psychosis at some point in their lives, (iii) were proficient in English, and (iv) were ≥18 years.

### 2.2 Measures

#### 2.2.1 Socio‐demographic and clinical information

Information on age, gender, ethnicity, education, and employment and clinical information was collected to assess study eligibility.

#### 2.2.2 Independent variable

##### Brief betrayal trauma survey (BBTS: Goldberg & Freyd, 2006)

The BBTS is a 12‐item self‐report measure that examines exposure to traumatic life experiences. Participants indicate if they have ever experienced a range of adverse life events both before and after the age of 18. We used nine items relating to childhood interpersonal trauma (i.e., items 3–10, including sexual, physical, or emotional abuse and witness of suicide/death/severe injury). The BBTS interpersonal trauma subscale has been used in survey studies with psychosis samples, including online research (Pearce et al., 2017), and has good validity and reliability (Goldberg & Freyd, 2006).

#### 2.2.3 Mediators

##### Psychosis attachment measure‐revised (PAM‐R: Pollard et al., 2020)

The PAM‐R is a 23‐item self‐report questionnaire of attachment patterns in psychosis adapted from the original PAM (Berry, Barrowclough, & Wearden, 2006) to include a disorganized attachment subscale. The PAM is a widely used measure (Berry et al., 2008). The PAM‐R has shown good psychometric properties (Pollard et al., 2020).

##### Dissociative experiences scale‐revised (DES‐II: Carlson & Putnam, 1993)

The DES‐II is a validated 28‐item self‐report questionnaire that measures dissociative experiences across three subscales: depersonalization and derealization (detachment), amnesia, and absorption (Carlson & Putnam, 1993). A total score of ≥30 has been used to identify clinical levels of dissociation (Carlson & Putnam, 1993).

##### Personality structure questionnaire (PSQ: Pollock et al., 2001)

The PSQ was used as measure of dissociative compartmentalization and is an eight‐item self‐report measure originally designed to measure the multiple self‐states model of identity disturbance (Ryle et al., 1997). The PSQ measures compartmentalization through dissociative shifts between different states of mind and has been correlated with dissociation, multiplicity, and identity disturbance (Pollock et al., 2001). It has demonstrated good psychometric properties in clinical and nonclinical samples (Bedford et al., 2009; Pollock et al., 2001) and has previously been used in psychosis samples (Taylor et al., 2019). Clinical levels of compartmentalization are suggested by a score of ≥26 (Berrios et al., 2016).

#### 2.2.4 Outcome variable

##### Self‐evaluation of negative symptoms (SNS; Dollfus et al., 2015)

The SNS is a 20‐item self‐report measure of negative symptoms. The measure has shown good psychometric properties in psychosis (Hervochon et al., 2018). SNS total scores of ≥7 have been identified as a threshold for screening clinical levels in people with schizophrenia (Dollfus et al., 2019). The total score was used for the main analysis. For exploratory analyses, the five subscales were combined to create negative symptom subdomains: expressive (diminished emotional range and alogia) and experiential (avolition, anhedonia, and social withdrawal).

#### 2.2.5 Confounders`

##### Community assessment of psychic experiences (CAPE: Stefanis et al., 2002)

The CAPE is a 42‐item self‐report measure designed to assess psychosis symptoms in general population samples and has demonstrated good psychometric properties (Konings et al., 2006; Stefanis et al., 2002). The depressive and positive frequency subscales were used.

### 2.3 Recruitment and procedure

Ethical approval was obtained from the University Research Ethics Committee. Participants were recruited via advertisements on mental health websites and forums and social media platforms (e.g., Twitter and Reddit). Local, national, and international mental health organizations, support groups, networks, and charities (e.g., hearing voices and paranoia groups) were contacted and requested to share the adverts online and/or with service users or members of their organization (face‐to‐face, emails, or letters). The survey commenced with a participant information sheet followed by the consent form. Participants were then invited to complete the sociodemographic questionnaire followed by the study measures presented in random order, with the trauma questionnaire (BBTS) placed in the middle to minimize participant distress. Participants were provided with a debrief sheet which included details of support services and were given the option to take part in a prize draw (shopping vouchers). Survey completion time ranged 30–45 min.

### 2.4 Analysis

Data were analysed using SPSS version 25 (IBM Corp, 2017). Preliminary analyses (*t‐*tests, ANOVAs, Pearson's correlations) examined relationships between sociodemographic data and study variables to determine statistically significant variables for inclusion in the mediation models. Bias‐corrected bootstrapping with 5,000 random samples corrected for nonnormal distributions (Cheung & Lau, 2007). Bonferroni correction accounted for multiple hypothesis testing (*p* < 0.001).

For mediation, bootstrapped partial correlations assessed the independence of the mediators, controlling for confounders positive and depressive symptoms in addition to socio‐demographic variables that correlated with the outcome in preliminary analyses. A series of mediation models were then conducted in AMOS v. 22 for SPSS (Arbuckle, 2006). Bias‐corrected bootstrapping with 5,000 random samples was used to examine the statistical significance of the indirect effect via the mediator, while accounting for nonnormal distributions (Preacher & Hayes, 2004).

Model 1 examined the direct/total effect of childhood interpersonal trauma on negative symptoms. Model 2 examined the regression coefficients between childhood interpersonal trauma and total negative symptoms, trauma and disorganized attachment, and disorganized attachment and total negative symptoms. Three subsequent models replaced the mediator, disorganized attachment, with avoidant attachment (Model 3), dissociation (Model 4), and compartmentalization (Model 5). For exploratory analyses, examining the separate pathways for the negative symptom subdomains, Models 1 to 5 were repeated for experiential (Models 6–10) and expressive (Models 11–15). Positive and depressive symptoms and age on the mediating and outcome variables were adjusted for in the mediation models. The DES‐II hearing voices item (item 27) was removed for mediation analyses. A final multiple mediation model including the variables found to mediate the relationship between childhood interpersonal trauma and total negative symptoms in the previous models was conducted. This allowed the simultaneous modelling of the direct effect and the relative indirect effects of the mediators on the outcome. Where statistically significant partial correlations were observed between the mediators, they were considered nonindependent and permitted to covary. Goodness‐of‐fit (GFI) statistics were estimated using the maximum likelihood estimation and the Bollen–Stine bootstrap (*n* = 5,000) procedure to correct for a multivariate nondistribution (Byrne, 2016). These included the following: Chi‐square test (*X* ^2^), Tucker–Lewis Index (TLI), Comparative Fit Index (CFI), GFI, and Root Mean Square Error of Approximation (RMSEA). Adequate fit of the model to the data is generally indicated by these fit indices: *X* ^2^ = <2, nonsignificant *p* value ≤0.05; TLI, CFI, and GFI= > 0.95; and RMSEA = <0.05 (Kline, 2015).

## 3 RESULTS

### 3.1 Sample and descriptive statistics (see Table 1)

**TABLE 1:** Sample and descriptive statistics (*n* = 242)

Characteristic	*M* (*SD*) /*N* (%)
Age (years)	33.17 (13.06)
Gender	
Male	154 (63.6)
Female	74 (30.6)
Prefer to self‐describe	14 (5.8)
Ethnicity	
White British	119 (49.2)
White other (European, Irish)	89 (36.8)
South or South East Asian	10 (3.3)
Black British/Caribbean/African	2 (0.8)
Mixed or other background	24 (9.9)
Employment	
Unemployed/receipt of sickness or disability benefits	79 (32.7)
Employed/self‐employed	91 (37.6)
Full time education	47 (19.4)
Retired/looking after family or home/other	25 (10.3)
Education	
Higher qualification (e.g., degree and teaching)	108 (44.6)
High school/A levels	59 (24.4)
Other qualifications	46 (19.0)
No qualifications	29 (12.0)
Self‐report psychosis	227 (93.8)
Current/previous diagnosis^a^	
Schizophrenia or paranoid schizophrenia	57 (23.6)
Schizoaffective	75 (31.0)
Schizophreniform	73 (30.2)
Delusional disorder	4 (1.7)
Depression with psychotic features	10 (4.1)
Bipolar with psychotic features	57 (23.6)
Brief psychotic disorder	15 (6.2)
Any other which includes psychotic experience	43 (17.8)
Other	47 (19.4)
Current antipsychotic medication	106 (43.8)
Current/previous mental health support or treatment for psychosis	220 (90.9)
Current inpatient	2 (0.8)
Previous inpatient	156 (64.5)
Current CMHT/EIS input	90 (37.2)
Previous CMHT/EIS input	162 (66.9)
BBTS childhood interpersonal trauma	12.89 (4.01)^b^
PAM‐R disorganised	1.49 (0.79)
PAM‐R avoidance	1.90 (0.72)
DES‐II dissociation	37.41 (24.07)^b^
PSQ compartmentalization	27.73 (6.92)^c^
SNS Total negative symptoms	21.19 (8.27)^c^
SNS expressive symptoms	8.21 (4.05)
SNS experiential symptoms	12.98 (13.00)
CAPE positive frequency	40.63 (11.98)^b^
CAPE depressive frequency	21.22 (5.23)

*Note*: SNS expressive symptoms include diminished emotional range and alogia; SNS experiential symptoms include avolition, anhedonia, and social withdrawal. Statistics are presented on raw data; Interquartile range (for skewed data): BBTS = 4.00; DES‐II = 32.87; PSQ = 6.00; SNS = 8.00; CAPE Positive = 12.25.

Abbreviations: BBTS, Brief Betrayal Trauma Survey, Childhood Interpersonal Trauma subscale; CAPE, Community Assessment of Psychic Experiences; CMHT, Community Mental Health Team; DES‐II, Dissociative Experiences Scale‐II; EIS, Early Intervention Services. PAM‐R, Psychosis Attachment Measure‐Revised; PSQ, Personality Structure Questionnaire; SNS, Self‐evaluation of Negative Symptoms.

^a^ Participants could select more than one diagnosis.

^b^ Data are positively skewed.

^c^ Data are negatively skewed.

A total of 242 participants were recruited and met the inclusion criteria. Of these, 227 (94%) reported they had a diagnosis of psychosis 220 (91%) reported receiving mental health support/treatment for psychosis. The sample was relatively young (*M* = 33.17), predominantly male (63.6%) and White British (49.2%) or White other (36.8%). Most participants reported a nonaffective schizophrenia‐spectrum diagnosis (86.4%).

Internal reliability scores were good‐to‐excellent for the measures: BBTS childhood interpersonal trauma (*α* = 0.80); PAM avoidance (*α* = 0.83) and disorganized (*α* = 0.90); DES total (*α* = 0.96); PSQ total (*α* = 0.84); SNS total (*α* = 0.87), expressive (*α* = 0.79) and experiential (*α* = 0.84); and CAPE positive (*α* = 0.90) and depressive (*α* = 0.84).

Of the 191 participants with data on the BBTS, 170 (89%) reported they had experience at least one form of childhood interpersonal trauma. Thirty‐eight percent had experienced sexual abuse, 38% physical abuse, 72% emotional abuse, and 28% had witnessed the death, suicide, or serious injury of a ‘very close’ person. Mean scores were above the advised clinical thresholds for the DES‐II (≥30), PSQ (≥26), and SNS (≥37), suggesting that the sample had clinically relevant levels of dissociation, compartmentalization, and negative symptoms.

Missing data ranged 13%–25% for study variables. Between‐group analyses revealed no statistically significant differences on study measures between participants with and without missing data. Missing data were managed by imputing the series mean at the subscale level; the total sample of 242 participants was included in the analyses providing sufficient power for mediation analyses with bootstrapping (Kline, 2015).

### 3.2 Preliminary analyses

Zero‐order and partial correlations are displayed in Table 2. Most of the hypothesized relationships in the models were statistically significant. Positive and depressive symptoms showed positive small to high associations with all study variables, with the strongest correlation observed between dissociation and positive symptoms. There were small negative correlations between age and dissociation and age and total negative symptoms. Disorganized attachment was lower in males (*M* = 1.4, *SD =* 0.69) than females (*M* = 2.6, *SD =* 0.68) (*t*(226)=−2.03, *p* = 0.043). There were no other significant associations between study measures and sociodemographic variables.

**TABLE 2:** Bootstrap‐corrected zero‐order correlations between study variables and partial correlations controlling for positive symptoms, depressive symptoms, and age (*n* = 242)

		1	2	3	4	5	6	7	8	9	10
1	BBTS childhood interpersonal trauma	‐	0.212*	0.092	0.156	−0.008	0.109	0.021	0.151	‐	‐
2	PAM‐R disorganised attachment	0.378*	‐	0.487*	0.256*	0.209*	0.337*	0.250*	0.304*	‐	‐
3	PAM‐R avoidant attachment	0.195	0.549*	‐	0.297*	0.123	0.521*	0.413*	0.449*	‐	‐
4	DES‐II dissociation	0.395*	0.478*	0.405*	‐	0.311*	0.213*	0.122	0.223*	‐	‐
5	PSQ compartmentalization	0.187	0.451*	0.256*	0.477*	‐	0.094	−0.005	0.148	‐	‐
6	SNS negative symptoms	0.291*	0.555*	0.580*	0.482*	0.354*	‐	0.797*	0.858*	‐	‐
7	SNS expressive symptoms	0.145	0.384*	0.472*	0.310*	0.170	0.810*	‐	0.374*	‐	‐
8	SNS experiential symptoms	0.330*	0.548*	0.522*	0.494*	0.405*	0.903*	0.479*	‐	‐	‐
9	CAPE positive symptoms	0.324*	0.449*	0.265*	0.649*	0.429*	0.451*	0.303*	0.453*	‐	‐
10	CAPE depressive symptoms	0.340*	0.583*	0.272*	0.514*	0.484*	0.522*	0.296*	0.564*	0.572*	‐
11	Age	0.004	−0.145	−0.146	−0.213*	−0.136	−0.212*	−0.181	−0.185	−0.166	−0.292

*Note:* Partial correlations to the right and zero‐order to the left of the diagonal.

Abbreviations: BBTS, Brief Betrayal Trauma Survey PAM‐R, Psychosis Attachment Measure‐Revised; CAPE, Community Assessment of Psychic Experiences; DES‐II, Dissociative Experiences Scale‐II (item 21 removed); PSQ, Personality Structure Questionnaire; SNS, Self‐evaluation of Negative Symptoms.

* Bonferroni adjusted statistical significance level: *p =* <0.001.

Statistically significant partial correlations were observed between most mediators; avoidant attachment did not correlate with compartmentalization. Partial correlations revealed a nonsignificant association between childhood trauma and the negative symptoms.

### 3.3 Mediation analyses

Tables 3, 4, 5 display the results of the mediation models.

**TABLE 3:** Mediation models to examine relationships between childhood interpersonal trauma, attachment, dissociation and negative symptoms, adjusted for age, and positive and depressive symptoms

Model	Effect	IV	DV	*β*	*BS SE*	*p*	*CI*	
Model 1.	Direct/total	Childhood trauma	Negative symptoms	0.11	0.07	0.101	−0.02	0.23
Model 2.	Direct	Childhood trauma	Disorganized attachment	0.18	0.06	0.001	0.07	0.31
	Direct	Disorganized attachment	Negative symptoms	0.34	0.07	<0.001	0.20	0.47
	Indirect	Childhood trauma	Negative symptoms	0.06	0.03	0.001	0.02	0.13
	Direct	Childhood trauma	Negative symptoms	0.05	0.07	0.491	−0.09	0.18
	Total	Childhood trauma	Negative symptoms	0.11	0.07	0.101	−0.02	−0.23
Model 3.	Direct	Childhood trauma	Avoidant attachment	0.11	0.07	0.141	−0.04	0.24
	Direct	Avoidant attachment	Negative symptoms	0.45	0.05	<0.001	0.35	0.54
	Indirect	Childhood trauma	Negative symptoms	0.05	0.03	0.127	−0.02	0.11
	Direct	Childhood trauma	Negative symptoms	0.06	0.06	0.280	−0.05	0.18
	Total	Childhood trauma	Negative symptoms	0.11	0.07	0.101	−0.02	0.23
Model 4.	Direct	Childhood trauma	Dissociation	0.19	0.06	0.001	0.07	0.31
	Direct	Dissociation	Negative symptoms	0.22	0.07	0.007	0.07	0.36
	Indirect	Childhood trauma	Negative symptoms	0.04	0.02	0.004	0.01	0.10
	Direct	Childhood trauma	Negative symptoms	0.07	0.06	0.286	−0.06	0.19
	Total	Childhood trauma	Negative symptoms	0.11	0.07	0.101	0.02	0.23
Model 5.	Direct	Childhood trauma	Compartmentalization	0.05	0.08	0.549	−0.10	0.20
	Direct	Compartmentalization	Negative symptoms	0.15	0.08	0.056	0.00	0.30
	Indirect	Childhood trauma	Negative symptoms	0.01	0.01	0.388	−0.01	0.05
	Direct	Childhood trauma	Negative symptoms	0.16	0.07	0.016	0.03	0.29
	Total	Childhood trauma	Negative symptoms	0.16	0.06	0.101	0.04	0.29

*Note*: Effects: the direct a path (IV ➔ mediator), the direct b path (mediator ➔ DV), the indirect c path (a*b); the direct c’ path (IV ➔ DV); the total effect (a*b + c′).

Abbreviations: BS SE, bootstrapped standard error; CI, 95% confidence interval; DV, dependent variable; IV, independent variable.

**TABLE 4:** Mediation models to examine relationships between childhood interpersonal trauma, attachment, dissociation and experiential symptoms, adjusted for age, and positive and depressive symptoms

Model	Effect	IV	DV	*β*	*BS SE*	*p*	*CI*	
Model 6.	Direct/total	Childhood trauma	Experiential symptoms	0.14	0.07	0.048	0.00	
Model 7.	Direct	Childhood trauma	Disorganized	0.18	0.06	0.001	0.07	0.31
	Direct	Disorganized	Experiential symptoms	0.29	0.07	<0.001	0.14	0.43
	Indirect	Childhood trauma	Experiential symptoms	0.05	0.02	<0.001	0.02	0.12
	Direct	Childhood trauma	Experiential symptoms	0.08	0.07	0.248	−0.06	0.22
	Total	Childhood trauma	Experiential symptoms	0.14	0.07	0.048	0.00	0.27
Model 8.	Direct	Childhood trauma	Avoidant attachment	0.11	0.07	0.141	−0.04	0.24
	Direct	Avoidant attachment	Experiential symptoms	0.37	0.06	<0.001	0.27	0.49
	Indirect	Childhood trauma	Experiential symptoms	0.04	0.03	0.118	−0.01	0.10
	Direct	Childhood trauma	Experiential symptoms	0.01	0.06	0.116	−0.02	0.22
	Total	Childhood trauma	Experiential symptoms	0.14	0.07	0.048	0.00	0.27
Model 9.	Direct	Childhood trauma	Dissociation	0.19	0.06	0.001	0.07	0.31
	Direct	Dissociation	Experiential symptoms	0.22	0.07	0.003	0.08	0.36
	Indirect	Childhood trauma	Experiential symptoms	0.04	0.02	0.002	0.01	0.10
	Direct	Childhood trauma	Experiential symptoms	0.10	0.07	0.144	−0.03	0.22
	Total	Childhood trauma	Experiential symptoms	0.14	0.07	0.048	0.00	0.27
Model 10.	Direct	Childhood trauma	Compartmentalization	0.05	0.08	0.549	−0.10	0.20
	Direct	Compartmentalization	Experiential symptoms	0.22	0.08	0.007	0.07	0.36
	Indirect	Childhood trauma	Experiential symptoms	0.01	0.02	0.455	−0.02	0.06
	Direct	Childhood trauma	Experiential symptoms	0.19	0.07	0.007	0.05	0.34
	Total	Childhood trauma	Experiential symptoms	0.20	0.07	0.002	0.07	0.34

*Note:* Effects: the direct a path (IV ➔ mediator), the direct b path (mediator ➔ DV), the indirect c path (a*b); the direct c’ path (IV ➔ DV); the total effect (a*b + c′).

Abbreviations: BS SE, bootstrapped standard error; *CI*, 95% confidence interval; DV, dependent variable; IV, independent variable.

**TABLE 5:** Mediation models to examine relationships between childhood interpersonal trauma, attachment, dissociation, and expressive symptoms, adjusted for age, and positive and depressive symptoms

Model	Effect	IV	DV	*β*	*BS SE*	*p*	*CI*	
Model 11.	Direct/Total	Childhood trauma	Expressive symptoms	0.03	0.07	0.601	−0.09	0.16
Model 12.	Direct	Childhood trauma	Disorganized	0.18	0.06	0.003	0.07	0.31
	Direct	Disorganized	Expressive symptoms	0.30	0.08	0.001	0.14	0.46
	Indirect	Childhood trauma	Expressive symptoms	0.06	0.02	0.001	0.02	0.12
	Direct	Childhood trauma	Expressive symptoms	0.21	0.06	0.758	−0.14	0.11
	Total	Childhood trauma	Expressive symptoms	0.03	0.06	0.597	−0.09	0.16
Model 13.	Direct	Childhood trauma	Avoidant attachment	0.11	0.07	0.141	−0.04	0.24
	Direct	Avoidant attachment	Expressive symptoms	0.40	0.07	<0.001	0.27	0.53
	Indirect	Childhood trauma	Expressive symptoms	0.04	0.03	0.119	−0.01	0.11
	Direct	Childhood trauma	Expressive symptoms	−0.01	0.06	0.922	−0.13	0.12
	Total	Childhood trauma	Expressive symptoms	0.03	0.06	0.597	−0.09	0.16
Model 14.	Direct	Childhood trauma	Dissociation	0.19	0.06	0.001	0.07	0.31
	Direct	Dissociation	Expressive symptoms	0.15	0.08	0.068	−0.01	0.30
	Indirect	Childhood trauma	Expressive symptoms	0.03	0.02	0.041	0.00	0.08
	Direct	Childhood trauma	Expressive symptoms	0.01	0.07	0.919	−0.12	0.14
	Total	Childhood trauma	Expressive symptoms	0.03	0.02	0.039	0.00	0.00
Model 15.	Direct	Childhood trauma	Compartmentalization	0.05	0.08	0.549	−0.10	0.20
	Direct	Compartmentalization	Expressive symptoms	0.01	0.08	0.972	−0.15	0.16
	Indirect	Childhood trauma	Expressive symptoms	0.00	0.01	0.895	−0.01	0.02
	Direct	Childhood trauma	Expressive symptoms	0.05	0.06	0.402	−0.07	0.18
	Total	Childhood trauma	Expressive symptoms	0.05	0.06	0.409	−0.07	0.18

*Note:* Effects: the direct a path (IV ➔ mediator), the direct b path (mediator ➔ DV), the indirect c path (a*b), the direct c′ path (IV ➔ DV), the total effect (a*b + c′).

Abbreviations: BS SE, Bootstrapped standard error; CI, 95% confidence interval; DV, dependent variable; IV, independent variable.

Model 1 shows no significant direct/total effect of childhood interpersonal trauma on total negative symptoms in psychosis. In Model 2, trauma predicted disorganized attachment, which predicted more severe negative symptoms. There was a significant indirect effect suggesting full mediation via disorganized attachment. There was no significant direct effect of childhood trauma on experiential or expressive symptoms (Models 6 and 11). However, significant indirect effects of trauma via disorganized attachment were observed for both experiential symptoms and expressive symptoms (Models 7 and 12). Childhood trauma did not directly predict avoidant attachment; there were direct effects of avoidant attachment on total negative symptoms, experiential symptoms, and expressive symptoms, but mediation via avoidant attachment was not observed for any of the negative symptom outcomes (Models 3, 8, and 13). Models 4 and 9 showed direct effects of childhood trauma on dissociation and dissociation on total negative symptoms and experiential symptoms. In both models, dissociation mediated the relationship between trauma and symptoms. These direct and indirect effects were not observed for dissociation and expressive symptoms (Model 14). There was no direct effect of childhood interpersonal trauma on compartmentalization, and hence, there was no indirect effect through this variable (Models 5, 10, and 15). The direct effects showed that compartmentalization predicted experiential symptoms (Model 10) but not total negative or expressive symptoms (Models 5 and 15).

### 3.4 Mediation analysis—Final model

**FIGURE 1:**
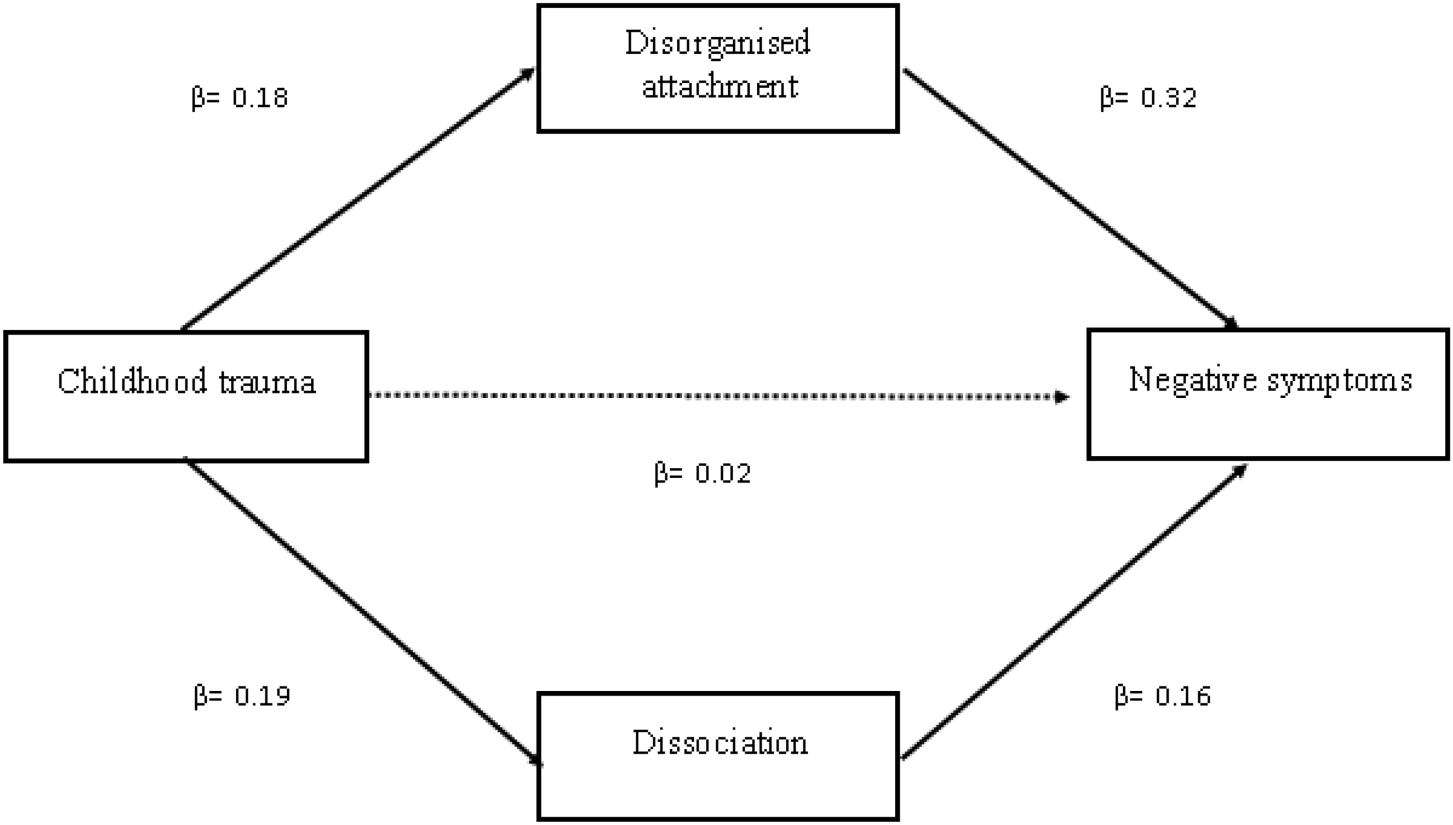
The final mediation model for total negative symptoms. *Note*: Standardized estimates presented for each path. Single headed arrows represent regression paths. Dotted line = effect not statistically significant

Based on the results considering the individual mediators, disorganized attachment and dissociation were included in a final model to predict total negative symptoms from childhood interpersonal trauma (see Figure 1). Parallel multiple mediation analysis included the mediators simultaneously in the model; positive and depressive symptoms and age were entered as covariates. Childhood trauma significantly predicted more disorganized attachment (*β =* 0.18, *p* = 0.001, *BS SE* = 0.06, 95% CI: 0.07, 0.31) and dissociation (*β =* 0.19, *p* = 0.001, *BS SE* = 0.06, 95% CI: 0.07, 0.31). Disorganized attachment (*β =* 0.32, *p* < 0.001, *BS SE* = 0.07, 95% CI: 0.17, 0.46) and dissociation (*β =* 0.16, *p* = 0.042, *BS SE* = 0.08, 95% CI: 0.01, 0.31) significantly predicted total negative symptoms. There was no direct effect of childhood trauma on total negative symptoms, suggesting that the effect was fully mediated by disorganized attachment and dissociation (*β =* 0.02, *p* = 0.752, *BS SE* = 0.07, 95% CI: *−*0.11, 0.14). However, the GFI suggested poor fit to the data (*X* ^2^ = 6.36(1), *p*= 0.012, Bollen–Stine bootstrap, *p* = 0.034; RMSEA = 0.149; GFI = 0.993; CFI = 0.990; TLI = 0.792). The model explained 40% of the variance in negative symptoms.

## 4 DISCUSSION

We examined the role of attachment and dissociative experiences in the pathway between childhood interpersonal trauma and negative symptoms in psychosis. Contrary to our primary hypothesis, childhood trauma did not independently predict negative symptoms. Adverse childhood experiences were frequently reported in our sample, with almost 90% of participants reporting at least one traumatic interpersonal event before the age of 18 years; these figures are higher than previous research in this area where significant associations have been reported (e.g., Sheinbaum et al., 2014; van Dam et al., 2014). Although bivariate analyses showed a positive association between trauma and negative symptoms, this was no longer statistically significant when controlling for age, depressive, and positive symptoms, suggesting that these confounders accounted for the relationship.

Hypotheses in relation to the psychological mediators between trauma and negative symptoms were partially supported. Consistent with our predictions, disorganized attachment and dissociative experiences (i.e., detachment, amnesia, and absorption) independently and fully mediated the relationship between childhood trauma and total negative symptoms. These findings were replicated when both the significant mediators were subsequently entered together into a combined mediation model, adjusting for positive and depressive symptoms and age. However, contrary to predictions, avoidant attachment and compartmentalization were not significant mediators.

These results build on previous research highlighting the mediating role of attachment in between trauma and negative symptoms (e.g., Sheinbaum et al., 2014) and positive symptoms (e.g., Pearce et al., 2017). They correspond to findings from one study showing that fearful attachment, but not avoidant attachment, was an important mediator between childhood trauma and subclinical negative symptoms (Sheinbaum et al., 2014). They are also consistent with the wealth of evidence suggesting that dissociation is an adaptive response to childhood trauma (Rafiq et al., 2018) and contributes to the evidence base suggesting clinically relevant associations between dissociative experiences and negative symptoms (Longden et al., 2020). Both negative symptoms and dissociation are suggested to function as self‐protective psychological mechanisms against adverse cognitive and affective stimulation related to trauma events (Vogel et al., 2011).

These early findings can be interpreted in line with attachment theories (Berry et al., 2017), suggesting that adverse childhood experiences are associated with disorganized attachment strategies, which in turn increase vulnerability to psychosis. Disorganized attachment has been shown to increase the risk of dissociation and fragmentation of self‐experience in response to trauma (Dutra et al., 2009). Dissociation has previously been conceptualized as a fear‐based disorganization’ (Liotti, 1992), and research suggests that disorganized attachment patterns in adulthood are more likely to lead to dissociative coping responses than other attachment patterns (Bucci et al., 2017; van Dam et al., 2014). The interrelationships between disorganized attachment and dissociation in their pathway to negative symptoms cannot be concluded based on the current study, using parallel mediation analysis and a cross‐sectional design. However, these early findings suggest that there is merit in undertaking further prospective research using sophisticated (serial) mediation models to determine causal pathways.

Consistent with previous research (Gumley, Taylor, et al., 2014), this study showed that higher levels of avoidant attachment predicted more severe negative symptoms. However, childhood trauma did not predict avoidant attachment, and therefore, significant mediation was not observed through this variable. Avoidant attachment patterns may be more related to neglectful childhood interpersonal experiences, which were not assessed in this study. Previous research has suggested that early traumatic abuse is more strongly related to disorganized attachment than other attachment patterns (Bucci et al., 2017). Individuals with avoidant attachment patterns are proposed to have at least one positive IWM of attachment relationships and more organized strategies which involve the deactivation of affects in response to distress (Liotti & Gumley, 2008). Disorganized attachment processes, on the other hand, are suggested to stem from contradictory and threatening caregiving responses which lead to negative IWMs of both the self and others (Liotti & Gumley, 2008). This may result in disorganized ways of regulating distress, whereby an individual may attempt to deactivate the attachment system to cope with fear (such as threat, rejection, or social exclusion) without the availability for better solutions (Liotti & Gumley, 2008).

The current study provides evidence to support potential psychological mechanisms involved in the pathway between childhood interpersonal trauma and negative symptoms. However, the model provided a poor fit to the data which suggests that other unmeasured variables may be important. Some of the unexplained variance in negative symptoms may be attributable to neurocognitive difficulties. Attachment may play an important role in the development of mentalization and social cognitive skills, which may heighten resilience to interpersonal stress and negative symptoms in psychosis (Ensink et al., 2016). Mentalization is a social cognitive process, by which one employs knowledge of the mental states of the self and others to understand social interaction (Fonagy et al., 2000). There is a growing evidence base for the impact of early trauma and attachment adversity on specific neurobiological pathways implicated in sensory‐affective processes (sensory and affect regulation) and higher‐order cognitive processes (mentalization and metacognition) that may underpin negative symptoms (Debbané et al., 2016). Other vulnerability factors could include affect regulation skills and beliefs about the self and others, which are associated with attachment processes and have been proposed in trauma models of psychotic experiences (Hardy, 2017). More specifically, social cognitive links between childhood abuse and disorganized attachment patterns may increase the risk of negative symptoms, where negative working models of the self and others and approach and avoidance regulation strategies contribute to emotional and cognitive disorganization (Sheinbaum et al., 2014). Attachment theory may offer an invaluable framework in conceptualizing the links between psychological and neurobiological processes in the development of negative symptoms (Griffiths & McLeod, 2020). Future longitudinal research is warranted to investigate further explanatory mechanisms in the relationships between childhood interpersonal trauma and adulthood expressions of disorganized attachment, dissociation, and negative symptoms in psychosis.

More work is needed to build a coherent developmental model of the mechanisms between childhood trauma and the clinical expression of negative symptoms in psychosis. The current study has highlighted the potential importance of adult attachment and dissociation. An avenue for further research is to examine the interactions between these psychological mechanisms and subclinical negative symptoms in the lead up to psychosis onset. Research suggests that the development of trait schizotypy increases the risk for transition to clinical expressions of psychosis, and that during adolescence, the development of negative and positive dimensions of schizotypy may interact to sustain expression and enhance the risk for psychosis onset (Debbané et al., 2014). Schizotypy has been conceptualized as a continuous personality dimension characterized by specific neurobiological, cognitive, and socioaffective organization. There is evidence that schizotypy traits are associated with specific endophenotypes and biomarkers, as well as neurocognitive and executive function impairments that underpin psychosis (for review, see Debbané et al., 2014). Negative schizotypy and symptoms such as asociality may arise from premorbid social interactions outside of caregiver attachment relationships such as with peers. For example, in school environments, children that express bizarre or odd behaviour may be more at risk of being bullied and withdrawing from social interactions with other children (Debbané et al., 2014). There may be advantage in evaluating the role of schizotypy as a developmental mediator in prospective studies evaluating the links between trauma and negative symptoms in psychosis.

This aim of this study was to understand the mechanisms linking trauma to negative symptoms in psychosis, which are under researched relative to positive symptoms. The results highlighted the confounding effects of positive and depressive symptoms in the link between childhood trauma and negative symptoms. Negative symptoms may occur prior to, in association with, or result from positive symptoms (Bobes et al., 2010; Rabinowitz et al., 2013). Negative symptoms may be secondary to positive or depressive symptoms. For example, an individual may become socially withdrawn after persecutory or paranoid ideas, or diminished emotional expression could be a coping strategy for someone who is unable to process overwhelming stimuli related to a psychotic episode. Negative symptoms may also precede positive symptoms and act as developmental mediators in the pathway to psychosis. Negative symptoms commonly occur before the onset of positive symptoms; 73% of patients reported negative symptoms before a psychotic episode and 20% reported them during the same month (An der Heiden et al., 2016). Further longitudinal research using sophisticated modelling may benefit from examining the interactions between negative, positive, and depressive symptoms and psychological mechanisms linking trauma and psychosis.

The exploratory analyses suggested that there may be differential pathways in the development of experiential versus expressive negative symptoms in psychosis (Strauss & Cohen, 2017). Disorganized attachment and dissociative experiences were significant mediators for experiential symptoms, but indirect effects were only observed via disorganized attachment (and not dissociation) for expressive symptoms. Dissociative experiences may, therefore, contribute or correspond to experiences involving social withdrawal and reduced interest or ability to experience pleasure but not expressive symptoms such as diminished expression or poverty of speech.

In this study, compartmentalization did not correlate with trauma and was not a significant mediator in the path between trauma and total negative symptoms. This suggests that compartmentalization may not necessarily develop in the context of early interpersonal trauma, which is inconsistent with previous findings in psychosis (Vogel et al., 2013). However, in the exploratory analyses, dissociative compartmentalization predicted experiential symptoms but not expressive or total negative symptoms. Reports from two previous studies (Vogel et al., 2009, 2013) suggest that poor identity integration may act as a defence mechanism against adverse mental events which increases the propensity to experience negative symptoms. However, the authors assessed total negative symptom scores and did not examine specific negative symptoms. Moreover, it is difficult to draw direct comparisons due to measurement differences, with the latter study using interview‐based measures of symptoms and dissociative compartmentalization.

Some methodological limitations warrant consideration. First, mediation models were examined using cross‐sectional methods. Thus, causal direction cannot be inferred; bidirectional associations between the psychological mediators and negative symptoms might exist. Second, controlling for depression may have been problematic given the overlap between depressive and negative symptoms, which may have partialled out meaningful variance in the outcome. Third, longitudinal research is needed to replicate the current findings and given the poor model fit, including additional variables and confounders that were not measured in this study (e.g., affect regulation, medication side effects, duration of psychosis). Fourth, online recruitment of participants may have introduced selection bias, for example, a bias towards participation of people with higher levels of initiation and motivation (and therefore struggling with negative symptoms) or those who were currently psychologically well. Previous reports suggest that online research may be biassed towards certain sociodemographic characteristics (Duggan & Brenner, 2013). The current sample was predominantly male; White, young, highly educated and around a third were unemployed. Evidence suggests that there is a higher prevalence of negative symptoms among men and the unemployed (Carbon & Correll, 2014; Kirkpatrick et al., 2001), and there is robust evidence to show that psychosis is higher among individuals from minority ethnic backgrounds particularly those of Black Caribbean ethnicity (Qaseem et al., 2015). Therefore, the sociodemographic profiles of our sample may not be representative of individuals with severe and persistent negative symptoms. Nevertheless, high levels of childhood trauma, insecure attachment, and clinically significant negative symptoms and dissociation were reported in the current study. Formal diagnostic criteria or clinical reports were not used to clarify psychosis, though research suggests online reports of psychosis are reliable (Moritz et al., 2013). Future research should use different sampling and data collection methods (e.g., in‐person recruitment) to minimize selection and reporter bias.

A final note on the measures: Research suggests that service users can accurately report most of their negative symptoms, regardless of their level of awareness, with the exception of alogia (Liraud et al., 2004). Clinician and service user ratings of alogia often differ (Bottlender et al., 2003). Additionally, the diminished emotional range of the SNS assesses emotions felt by the respondent which may represent a distinct dimension and require separate assessment from more observational measures of blunted affect such as unchanging facial expression, paucity of expressive gestures and poor eye contact (Dollfus et al., 2015). It is important to consider this when interpreting the findings related to the self‐report assessment of the ‘expressive’ domain and drawing comparisons with studies using standardized clinical interviews. The lack of precision when measuring negative symptoms may lead to observed associations with experiential symptoms or social withdrawal rather than other types of negative symptoms. The PAM‐R (Pollard et al., 2020) is a measure of current adult attachment patterns and may not reflect attachment patterns in childhood or capture unconscious or affective attachment representations. Self‐report assessments can underestimate attachment, arising from the activation of the attachment system whereby people who are avoidant of attachment may be more likely to rate attachment as secure (Gumley, Taylor, et al., 2014). The DES (Carlson & Putnam, 1993) and PSQ (Pollock et al., 2001) may have comprised overlapping constructs, and the validity of the PSQ as a measure of dissociative compartmentalization in psychosis requires further investigation. Finally, the measures used may be susceptible to the confounding effects of individual variables including personality and symptomatic expression. Further research is warranted using standardized assessment tools to determine whether specific types of dissociation lead to negative symptoms or symptom subtypes in psychosis.

This study provides early findings suggesting that there may be benefit in exploring the relationships between childhood interpersonal trauma, attachment, and dissociation in individualized assessments and formulations for negative symptoms. Research has increasingly moved towards developing more specialized psychological interventions for negative symptoms to improve the effect sizes of standard models that traditionally focus on psychosis symptoms simultaneously (Lutgens et al., 2017). The current findings suggest that there may be value in psychological assessment of negative symptoms in clinical practice, considering the experiences of specific symptoms from the service user viewpoint (Dollfus et al., 2019); for example, exploring how experiential symptoms such as low social drive or motivation may have developed, how they may interact with dissociative experiences and attachment patterns, and the ways in which they may impact on current symptom‐related distress.

The finding of an association between insecure disorganized and avoidant attachment patterns and negative symptoms has implications for psychological therapy (Mallinckrodt et al., 1995, 2009). Individuals exhibiting an avoidant or disorganized attachment pattern in the context of negative symptoms may experience the therapeutic relationship to be a source of stress or threat, which may lead to unhelpful emotion regulation strategies and subsequent emotional avoidance or social withdrawal (Griffiths & McLeod, 2020). Therapists may consider how to strengthen the alliance and provide a secure base for positive attachment representations to develop (Taylor et al., 2015).

Trauma‐informed interventions can support individuals with negative symptoms and avoidant or disorganized attachment to manage emotion dysregulation and dissociative responses through grounding and soothing techniques. Mindfulness‐based approaches may be helpful for dissociation in response to attachment trauma, such as supporting the client to increase conscious awareness to the present moment and internal and external experiences (Brown et al., 2007). Person‐centred approaches may include identifying dissociative reactions such as shifts in affective and cognitive states, exploring triggering events, and using distraction, grounding, and cognitive restructuring techniques (Kennerley, 1996).

## 5 CONCLUSION

Negative symptoms represent an unmet need and are related to adverse psychosocial outcomes in psychosis. There is a lack of effective interventions for negative symptoms and their underlying psychological mechanisms remain poorly understood. This study is the first to show that disorganized attachment and dissociative experiences may be important mediators in the pathways between childhood trauma and negative symptoms. Avoidant attachment and compartmentalization were independent predictors of negative symptoms but were not associated with early interpersonal trauma. There was evidence to suggest that dissociative mechanisms may be more important for experiential negative symptoms, involving social withdrawal, avolition, and anhedonia, rather than expressive symptoms, involving diminished emotional expression and alogia. These results support a dimensional symptom‐specific approach to assessment and formulation which may improve our understanding of and inform specialized interventions for negative symptoms in psychosis. Further longitudinal studies are needed to replicate these findings and test theoretical models relating to explanatory and mediating mechanisms.

## CONFLICT OF INTEREST

None.

## Data Availability

Data are available on request from the authors.
